# Comparative Analysis of Patient-Reported Outcome Measures in Manual Small-Incision Cataract Surgery Versus Phacoemulsification for Brown Cataracts

**DOI:** 10.7759/cureus.75260

**Published:** 2024-12-07

**Authors:** Anjali Goel, Matuli Das, Saswati Sen

**Affiliations:** 1 Department of Ophthalmology, Kalinga Institute of Medical Sciences, Bhubaneswar, Bhubaneswar, IND

**Keywords:** brown cataract, catquest questionnaire, phacoemulsification, prom study, sics

## Abstract

Objective

The objective of this study is to compare patient-reported outcome measures using the Catquest Questionnaire in patients undergoing phacoemulsification (Phaco) versus manual small-incision cataract surgery (MSICS).

Materials and methods

This descriptive cross-sectional study included patients aged 40 years and older with cataracts classified as nuclear sclerosis (NS) grade 3 or higher. Demographic details were recorded and a comprehensive ophthalmological exam was done. All patients were operated on by the same surgeon, with 41 undergoing MSICS and 40 undergoing Phaco. Monofocal intraocular lenses were implanted in all cases. Responses to the Catquest questionnaire were collected preoperatively and at six weeks postoperatively. The questionnaire, validated in the Odia language, was provided to patients in both Odia and English.

Results

Out of 81 patients, 32 underwent their first eye surgery while 49 had their second eye surgery. Both Phaco and MSICS procedures showed significant visual acuity improvement. Mean visual acuity improved from 1.19 to 0.37 in the right eye and from 0.74 to 0.35 in the left eye, with p-values <0.001. Nearly all patients experienced better near vision postoperatively, with 45 (97.8%) of right eyes and 34 (100%) of left eyes achieving near vision between N6 and N8. One Phaco patient with age-related macular degeneration had near vision limited to N10. In a few areas, such as carrying out hobbies, doing needlework, and overall vision satisfaction, patients in the MSICS group patients did better than Phaco group. Response to other questions showed similar responses in both the Phaco and MSICS groups.

Conclusion

Cataract surgery irrespective of procedure improves overall vision-specific functioning and quality of life. MSICS is often preferred over Phaco for its speed, cost-effectiveness, and lower technological dependence, especially for brown cataracts and bulk surgeries. The choice between MSICS and Phaco should depend on patient needs, preoperative counseling, surgeon expertise, and resources.

## Introduction

Cataract is the leading cause of avoidable blindness globally, with surgical techniques evolving to enhance visual outcomes and patient satisfaction [1]. Initially, cataracts were treated by couching, a method dating back to the fifth century BC where a needle displaced the cataractous lens. Modern surgeries have progressed to extracapsular cataract extraction (ECCE), primarily performed using manual small-incision cataract surgery (MSICS) or phacoemulsification (Phaco) [2].

MSICS involves prolapsing the lens nucleus through a scleral incision whereas Phaco, introduced in 1967, uses an ultrasound-driven needle to emulsify the lens through a smaller corneal incision, offering a more advanced technique. Since then the technique has developed and is used effectively for hard cataracts as well [3].

Recent studies have shown that MSICS, once deemed inferior, is cost-effective and nearly as effective as Phaco [4]. Surgical outcomes are traditionally assessed through objective measures like visual acuity and contrast sensitivity. However, patient-reported outcome measures (PROMs) provide insight into patient satisfaction and quality of life post-surgery [5,6].

Recent years have highlighted the importance of incorporating the patient's perspective in ophthalmology, influencing our understanding of disease impact and intervention effectiveness. There's been a shift from traditional metrics (e.g., visual acuity, intraocular pressure (IOP)) to those reflecting patient and provider priorities (e.g., symptoms, quality of life, convenience, cost). PROMs capture these crucial aspects. Patient satisfaction is particularly variable in cases of brown cataracts (nuclear sclerosis (NS) grade 3 and above), following both Phaco and MSICS procedures. Standardized questionnaires like the Catquest and Cat-PROMs are used to evaluate the qualitative impact on daily life and independence [7].

This study aims to compare MSICS and Phaco using the Catquest questionnaire [8] to assess real-life implications and patient satisfaction, informing improvements in preoperative counseling, surgical techniques, and post-operative care.

## Materials and methods

This is a descriptive cross-sectional study, which was conducted after obtaining institutional ethical clearance (KIIT/KIMS/IEC/952/2022). The study complied with the principles of the Declaration of Helsinki. The study was conducted over a two-year period, from 2022 to 2024, in the Department of Ophthalmology at a tertiary care center in Eastern Odisha, India.

By keeping an effect size of 0.40, 5% significance level, 95% confidence interval, and 80% power, the minimum required sample size was determined to be 78 (39 per group). Patients of the age group above 40 years with cataracts of NS grade 3 and above were included in the study. Exclusion criteria comprised patients who refused to consent, those with significant vision loss due to other eye diseases (such as optic nerve damage or retinal diseases), and individuals with pre-existing systemic or ocular conditions affecting vision beyond cataracts.

Eighty-one patients aged 40 years and above with operable senile cataracts (NS grade 3 or higher, based on the Lens Opacities Classification System III (LOCS III)) underwent surgery, as categorized by the criteria established by Chylack et al. [9]. The participants were divided into two groups: 41 underwent MSICS and 40 underwent Phaco. Informed consent was obtained from all patients, and their demographic data, clinical history, and examination results were meticulously recorded. Anterior and posterior segments were evaluated as per standard protocols and preoperative assessments including demographic data, intraoperative events, and postoperative assessment data were collected along with responses of the patients preoperatively and at six weeks postoperatively. The Catquest questionnaire which was available in the public domain was also given preoperatively and at six weeks postoperatively. The Catquest questionnaire had questions in three sections. The first section has six questions about how vision influences daily life routine activities. The second section has five questions, which are graded on the basis of difficulty in performing tasks graded from extreme difficulty to least difficulty. The third section has six questions pertaining to the patient’s general health. The Catquest questionnaire used in this study was internally validated in the local language, Odia (Appendix A).

Data collected were compiled in the Microsoft Excel worksheet (Microsoft® Corp., Redmond, WA, USA) and analyzed using Epi-Info software (version 7.2.5.0; Centers for Disease Control and Prevention (CDC), Atlanta, USA). Data was expressed as frequency and percentages for categorical variables and as mean±SD for continuous variables. The chi-square test was used to measure association. Student's t-test and Mann-Whitney test were used as tests of significance for group comparison, as applicable. A p-value of ≤ 0.05 was considered statistically significant.

## Results

In our study, the average age group was 68 years in the Phaco group and 67.13 years in the MSICS group. Demographic data that was collected showed no significant difference between males and females. Also, there was no significant statistical difference in the income and education of patients between the two surgical groups. The details are given in Table 1.

**Table 1:** Demographic data Phaco: phacoemulsification; MSICS: manual small-incision cataract surgery

Variables	Male n(%)	Female n(%)	Total (N=81)	P-value
Age				
<60 years	6 (17.1%)	10 (21.7%)	16 (19.7%)	0.573
>60 years	29 (82.8%)	36 (78.2%)	65 (80.2%)	
Education				
Graduate (12th std pass)	20 (57.1%)	21 (45.6%)	41 (50.6%)	0.352
Postgraduate	15 (42.8%)	25 (54.3%)	40 (49.3%)	
Income				
Above poverty line	26 (74.2%)	30 (65.2%)	56 (69.1%)	0.460
Below poverty line	9 (25.7%)	16 (34.7%)	25 (30.8%)	
Surgical procedure				
Phaco	19 (54.2%)	21 (45.6%)	40 (49.3%)	0.498
MSICS	16 (45.7%)	25 (54.3%)	41 (50.6%)	
Total	40 (49.3%)	41 (50.6%)		

Visual acuity improved in both groups after cataract surgery (p<0.001) with no statistically significant difference between the two surgeries for distance visual acuity or near visual acuity. In Phaco, one patient did not show improvement for near vision up to 6/6, which was most probably attributed to age-related macular degeneration (ARMD) findings in the fundus. The IOPs, both preoperative and postoperative, showed no significant fluctuation (Table 2). The average Phaco time was 21.3 minutes, with an average Phaco power of 31.15.

**Table 2:** Comparison of objective parameters MSICS: manual small-incision cataract surgery; LogMAR: logarithm of the minimum angle of resolution

	Surgery	Phacoemulsification		MSICS		P-value
Parameter		Right eye	Left eye	Right eye	Left eye	0.096
Visual acuity distance (in LogMAR)	Pre-op	1.06 ±0.75	0.71±0.56	1.32±0.67	0.76±0.24	
	Post-op	0.34±0.45	0.29±0.47	0.40±0.28	0.41±0.39	
Intraocular pressure (in mmHg)	Pre-op	13.7±2.63	14.2±2.24	16.41±2.0	13.98±2.2	<0.001
	Post-op	13.55±2.0	13.98±2.2	15.8±2.2	16.5±2.7	

Table 3 demonstrates the comparison of subjective parameters between the two groups.

**Table 3:** Comparison of subjective parameters MSICS: manual small-incision cataract surgery

Surgery		Phacoemulsification					MSICS					P-value
Parameters (n(%))		Extreme	Much	Some	No	Cannot say	Extreme	Much	Some	No	Cannot say	
Reading newspaper print	Pre-op	0	2(5%)	31(77.5%)	5(12.5%)	2(5%)	0	0	17(41.5%)	0	24(58.5%)	<0.001
	Post-op	0	0	12(30%)	26(65%)	2(5%)	0	0	0	25(61%)	16(39%)	<0.001
Recognizing faces	Pre-op	0	0	9(22.5%)	31(77.5%)	0	0	0	24(59.5%)	17(41.5%)	0	<0.001
	Post-op	0	0	0	40(100%)	0	0	0	0	41(100%)	0	-
Doing needlework	Pre-op	0	2(5%)	5(12.5%)	31(77.5%)	2(5%)	0	0	1(2.5%)	8(20%)	32(77.5%)	0.016
	Post-op	0	0	1(2.5%)	8(20%)	31(77.5%)	0	0	0	0	41(100%)	0.006
Pursuing hobbies	Pre-op	0	0	4(10%)	7(17.5%)	39(72.5%)	0	0	0	0	41(100%)	0.001
	Post-op	0	0	0	11(27.5%)	39(72.5%)	0	0	0	0	41(100%)	<0.001
Problems in daily life due to present vision	Pre-op	0	1(2.5%)	33(82%)	6(15%)	0	0	0	41(100%)	0	0	0.020
	Post-op	0	0	0	40(100%)	0	0	0	0	41(100%)	0	-

**Figure 1:**
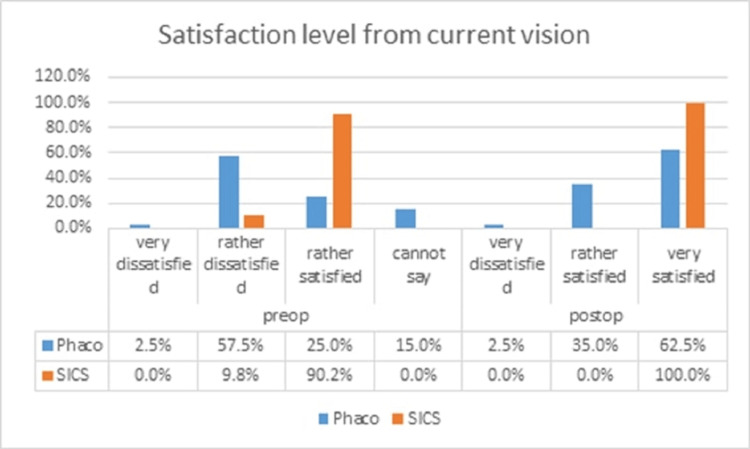
Satisfaction level from current vision Phaco: phacoemulsification; SICS: small-incision cataract surgery

In both Phaco and MSICS groups, the percentage of participants reading the newspaper increased postoperatively. A greater number of MSICS patients began reading two newspapers daily, demonstrating an increase in reading ability postoperatively. Most patients who answered some difficulty in reading newspaper print preoperatively answered no difficulty postoperatively. A few patients in the Phaco group showed an increase in the number of hours spent watching TV, rising from one hour per day to several hours per day postoperatively. Phaco patients who initially answered “yes” to experiencing difficulty with shopping independently reported improvement postoperatively and had no difficulty shopping by themselves due to vision. Preoperatively, patients in both groups reported some difficulty in recognizing the faces of people they met. However, postoperatively, all patients reported no difficulty, indicating an improvement in vision following surgery. Phaco patients who reported some difficulty preoperatively in doing needlework, answered “no difficulty” postoperatively, indicating an improvement in their ability to perform the task. In the Phaco group, four patients (10%) reported some difficulty and seven patients (17.5%) reported much difficulty preoperatively. Postoperatively, 11 patients (27.5%) reported much difficulty, indicating that for some patients, doing hobbies became more difficult after Phaco surgery. Most patients in both groups reported some difficulty preoperatively, but postoperatively, all patients reported no difficulty with vision in daily life. Regarding satisfaction with their current vision, preoperatively, patients in the Phaco group reported being rather dissatisfied, while patients in the MSICS group were generally rather satisfied. Postoperatively, most patients in the Phaco group reported being rather satisfied, while all patients in the MSICS group expressed being very satisfied with their vision (Figure 1).

## Discussion

In our study, which included 81 participants divided into two groups - Phaco (n=40) and MSICS (n=41) - we observed that the mean age of patients in the Phaco group was 68 years, while the MSICS group had a mean age of 67.13 years. This age difference was not statistically significant (p=0.691), consistent with findings from Singh et al. [5] who reported no significant differences in age, gender, or preoperative visual acuity between the two groups (p=0.09).

In contrast, Lundström et al. [10] found that patients older than 85 years experienced less improvement in visual function compared to younger patients, suggesting that age might impact visual outcomes. Similarly, Rönbeck et al. [11] observed that younger patients with low preoperative visual acuity, no ocular comorbidities, and mild postoperative residual myopia had significantly better subjective visual function (p<0.001).

In our study, both Phaco and MSICS groups showed significant improvements in best corrected visual acuity (BCVA) postoperatively. Specifically, BCVA in the right eye improved from 1.19 preoperatively to 0.37 postoperatively, and in the left eye from 0.74 to 0.35 (p<0.001 for both). There was no significant difference in visual acuity outcomes between the two groups (p>0.05), aligning with results from Bhargava et al. [12] and Gogate et al. [13]. However, Cook et al. [14] and Yorston and Abiose [15] found that Phaco showed better long-term visual outcomes compared to MSICS. In addition, the Phaco time and power used in the present study was less than that in comparison to other studies like Gonen et al. [16] and Fernández-Muñoz et al. [17] on Phaco of brown cataracts.

Our study also examined gender differences in visual outcomes and found no significant disparity between male and female patients, consistent with Singh et al. [5]. This finding contrasts with Lundqvist and Mönestam [18] who reported that female patients perceived their visual function worse than male patients and had significantly lower Visual Function Index-14 (VF-14) scores before and after surgery.

Regarding IOP, we noted a decrease in both Phaco and MSICS groups postoperatively. The reduction was more pronounced in patients with higher baseline IOP, aligning with the study by Sengupta et al. [19], which showed similar IOP reductions in both surgical techniques and a greater decrease in IOP correlated with higher baseline levels.

Using the Catquest questionnaire to assess various aspects of vision-related daily life, we found improvements in activities such as reading newspapers, shopping, and watching television. However, difficulties in hobbies persisted for some patients, particularly in the Phaco group. These results are similar to those reported by Elliott et al. [20] who found significant improvements across multiple vision-related domains post-surgery.

Patient satisfaction improved markedly, with 100% of patients in both groups reporting "no difficulty" in vision-related activities postoperatively. This improvement is consistent with findings from Colin et al. [21], who reported high levels of satisfaction with surgery, and Do et al. [22], who found that patients scored health-related quality of life highly postoperatively. Schlenker et al. [23] found that both preoperative and postoperative visual acuity, as well as questionnaire results from the Catquest-9SF, were significantly associated with the appropriateness of cataract surgery. They highlighted the importance of combining visual acuity and PROMs in prioritizing patients. Another study by Schlenker et al. [24] also identified significant factors such as BCVA, night-driving difficulty, and daily tasks in determining appropriateness.

Our study found that 17 (42.5%) of Phaco patients and 37 (90.2%) of MSICS patients had additional medical conditions. Despite this, their visual improvements were similar to those of patients without comorbidities. This contrasts with Yong et al. [25], who identified systemic and ocular comorbidities as risk factors for poorer postoperative visual outcomes.

Lundström and Pesudovs [26], in a multicentric study using the Catquest questionnaire, reported that 90.9% of patients experienced a benefit from cataract surgery. The framework of the Catquest questionnaire effectively captured different levels of benefit, aligning closely with patients' global ratings of their vision and the visual acuity achieved post-surgery. Similarly, in our study, we observed that all patients who experienced improved visual acuity after surgery reported no difficulties with their daily activities when questioned. This suggests that the Catquest questionnaire, like its successor Catquest-9SF, provides a reliable framework for assessing subjective improvements in patients' vision.

To summarize, our study demonstrates that relying solely on clinical metrics might underestimate the overall benefits of cataract surgery. Patients with initially very poor visual acuities not only achieved significant improvements in visual acuity but also reported enhanced daily life activities. Desai et al. [27] and Nijkamp et al. [28] also highlighted the importance of considering both clinical outcomes and patient-reported outcomes to fully understand the impact of cataract surgery.

Overall, cataract surgery significantly enhances vision-specific functioning and quality of life, including for patients with comorbid eye diseases, particularly in their early stages. Second-eye cataract surgery typically offers greater visual improvement compared to first-eye surgery. The shift towards second-generation, Rasch-validated PROMs has shown notable gains in visual function, with improved measurement precision compared to earlier PROM devices.

Limitations

The short duration of the study and small sample size were a few of the limitations of the present study. First-eye versus second-eye surgeries were also not assessed separately. Objective vision changes related to endothelial cell loss in Phaco were also not accounted for due to lack of instrumentation. As the focus was more on subjective parameters, surgically induced astigmatism was not considered for comparison.

## Conclusions

PROM studies indicate that both MSICS and Phaco offer significant improvements in visual outcomes and quality of life for patients undergoing cataract surgery. MSICS is valued for its cost-effectiveness and minimal technological requirements, while Phaco, though more expensive and technology-intensive, has benefits such as faster recovery and fewer chances of astigmatism. Both techniques provide comparable visual acuity results. MSICS is a viable choice, especially in resource-limited settings with the need for bulk surgeries where advanced technology might be inaccessible. Phaco, on the other hand, is the preferred method in developed countries.

The decision between MSICS and Phaco should be guided by individual patient needs, preoperative counseling to manage expectations, surgeon expertise, and available resources. Ongoing long-term PROM studies are essential to fully understand the sustained benefits and patient preferences for these cataract surgery techniques.

## Appendices

Appendix A - Catquest questionnaire

A.    Do you normally read a newspaper? No 0 Yes 0

·       If yes, do you normally read

o   One newspaper a week at the most 0

o   One newspaper a day (approx.) 0

o   Several newspapers a day 0

·       If you normally do not read a newspaper, is it only because of poor vision?

o   Yes 0

o   No 0

B.    Do you normally buy your nondurable goods yourself or regularly make other purchases?

o   No 0

o   Yes 0

·       If yes, do you normally shop

o   Once a week 0

o   at the most 2-4 times a week (approx.) 0

o   Daily 0

If you normally do not shop yourself, is it only because of poor vision?

o   Yes 0

o   No 0

C.    Do you normally take a walk outside on your own or with company?

o   No, never 0

o   Yes 0 

·       If yes, how often?

o   Once a week   0

o   at the most 2-4 times a week (approx.)  0

o   Daily 0

·       If you never take a walk outside, is it only because of poor vision?

o   Yes 0

o   No 0

D.    Do you normally do needlework, woodwork, embroidery, or other handicrafts?

o   No, never 0

o   Yes 0

·       If yes, how often?

o   Once a week

o   at the most 2-4 times a week (approx.)

o   Daily

·       If you normally do not do needlework or other handicrafts, is it only because of poor vision?

o   Yes 0

o   No 0

E.     Do you normally watch television?

o   No, never 0

o   Yes 0

·       If yes, how often?

o   Once a week 0

o   at the most One hour daily (approx.) 0

o   Several hours daily  0

·       If you normally do not watch television, is it only because of poor vision?

o   Yes 0

o   No 0

F.     Do you have another hobby or leisure activity that you would like to do?

o   No 0

o   Yes 0

 Hobby/activity _________ _ (Fill in the name of the activity.)

 If you have answered yes to this question, how often do you perform this activity?

o   Once a week  0

o   at the most 0 2-4 times a week (approx.) 0

o   Daily 0

• When you answer the next set of questions, A through E, try to think only of problems related to your vision. We understand it may be difficult to decide the exact effects of vision if you have other problems such as arthritis or dizziness. Still, try to answer how you think your vision affects your ability to carry out the following activities. To describe your difficulties, we use one of three possible answers: extreme difficulty, much difficulty, and some difficulty. View the three possible answers as three equally sized grades on a scale from the most difficult to the least difficult as shown below: Most difficult ....... / ....... / ...... Least difficult

                Extreme    Much    Some  (difficulty)

A.    Because of your vision, do you have difficulty with the following activities? If yes, how much? Put only one check mark on each row in the box you think most reflects reality.

                                                                 Yes, extreme   Yes, much     Yes, some     No, no         Cannot

                                                                        difficulty      difficulty      difficulty     difficulty         say

 Read newspaper print                                         0                   0                0                  0                  0

 Recognize the faces of people you meet         0                   0                0                  0                  0

 See the prices of goods when you shop           0                   0               0                  0                  0

 See to walk on uneven ground                          0                    0               0                   0                 0

 See to do needlework, etc.                                0                    0                0                   0                 0

 Read television text                                            0                    0                 0                   0                 0

 See to carry out the activity/                            0                     0                 0                  0                  0

hobby you named previously

Does your present vision in any way give you problems in your daily life?

o   Yes, extreme difficulty 0

o   Yes, much difficulty 0

o   Yes, some difficulty 0

o   No, no difficulty 0

o   Can't say 0

B.    Are you satisfied or dissatisfied with your present vision?

Very dissatisfied 0

Rather dissatisfied 0

Rather satisfied 0

Very satisfied 0

Cannot say 0

C.    Different lighting conditions (darkness, rain) can sometimes influence one's vision, producing visual disturbances such as glare or dazzling. Do you think that headlights, lamps, sunlight, and other lights reduce your vision more often now than before?

o   No, never 0

o   Yes 0

·       If yes, does it give you

o   Extreme difficulty 0

o    Much difficulty     0

o   Some difficulty      0

o    No difficulty          0

o    Cannot say            0

D.    In persons with cataract, great visual differences between the two eyes can occur. This can lead to poor depth perception so that you may, for example, spill when pouring out liquids. If one eye is already operated on, one can experience great differences in clarity and color between the two eyes. Do you experience visual disturbances from any of the above named differences between the two eyes?

o   No 0

o   Yes 0

·       If yes, does it give you

o   Extreme difficulty 0

o    Much difficulty     0

o   Some difficulty      0

o    No difficulty          0

o    Cannot say            0

• The following questions concern your general health:

A. Do you have any illness for which you take medicine regularly?

o   No  0

o   Yes, one illness  0

o   Yes, more than one illness  0

o   Cannot say  0

B. Do you have help in your home (other than from those living in your home)?

o   Yes, help from a friend/relative   0

o   Yes, home help    0

o   Yes, from staff at the aged persons home/nursing home/hospital    0

o   Cannot say   0

·       If you have daily help, how many hours per day? _____ hours/day

·       If you have help each week, how many hours per week? _____ hours/week

C. Do you have subsidized travel by taxi?

o   No 0

o   Yes 0

D. Do you have employment?

o   No 0

o   Yes 0

·       If yes, are you off sick?

o    No 0

o   Yes 0

E. Do you live alone?

o   No 0

o   Yes 0

F. Have you driven a car during the past 12 months?

o   No 0

o   Yes 0

·       If yes, what is the situation at present?

o   Drive both during day and at night? 0

o    Drive only in daylight 0

o    Have given up driving because of poor vision 0

o    Have given up driving for other reasons 0

G. If you still drive a car, does your vision give you difficulty while you are driving?

o   Yes, extreme difficulty 0

o   Yes, much difficulty 0

o   Yes, some difficulty 0

o   No, no difficulty 0

o   Cannot say 0

## References

[1] Resnikoff S, Pascolini D, Etya'ale D, Kocur I, Pararajasegaram R, Pokharel GP, Mariotti SP (2004). Global data on visual impairment in the year 2002. Bull World Health Organ.

[2] Stürmer J (2009). Cataracts - trend and new developments [Article in German]. Ther Umsch.

[3] Abdelmotaal H, Abdel-Radi M, Rateb MF, Eldaly ZH, Abdelazeem K (2021). Comparison of the phaco chop and drill-and-crack techniques for phacoemulsification of hard cataracts: a fellow eye study. Acta Ophthalmol.

[4] Gogate P, Deshpande M, Nirmalan PK (2007). Why do phacoemulsification? Manual small-incision cataract surgery is almost as effective, but less expensive. Ophthalmology.

[5] Singh SK, Winter I, Surin L (2009). Phacoemulsification versus small incision cataract surgery (SICS): which one is a better surgical option for immature cataract in developing countries?. Nepal J Ophthalmol.

[6] Wiklund I (2004). Assessment of patient-reported outcomes in clinical trials: the example of health-related quality of life. Fundam Clin Pharmacol.

[7] Sparrow JM, Grzeda MT, Frost NA (2018). Cat-PROM5: a brief psychometrically robust self-report questionnaire instrument for cataract surgery. Eye (Lond).

[8] Lundström M, Roos P, Jensen S, Fregell G (1997). Catquest questionnaire for use in cataract surgery care: description, validity, and reliability. J Cataract Refract Surg.

[9] Chylack LT Jr, Wolfe JK, Singer DM (1993). The lens opacities classification system III. The Longitudinal Study of Cataract Study Group. Arch Ophthalmol.

[10] Lundström M, Stenevi U, Thorburn W (2000). Cataract surgery in the very elderly. J Cataract Refract Surg.

[11] Rönbeck M, Lundström M, Kugelberg M (2011). Pancreatic cancer metastasis to choroid. Ophthalmology.

[12] Bhargava R, Kumar P, Sharma SK, Kumar M, Kaur A (2015). Phacoemulsification versus small incision cataract surgery in patients with uveitis. Int J Ophthalmol.

[13] Gogate P, Optom JJ, Deshpande S, Naidoo K (2015). Meta-analysis to compare the safety and efficacy of manual small incision cataract surgery and phacoemulsification. Middle East Afr J Ophthalmol.

[14] Cook C, Carrara H, Myer L (2012). Phaco-emulsification versus manual small-incision cataract surgery in South Africa. S Afr Med J.

[15] Yorston D, Abiose A (2001). Cataract blindness - the African perspective. Bull World Health Organ.

[16] Gonen T, Sever O, Horozoglu F, Yasar M, Keskinbora KH (2012). Endothelial cell loss: biaxial small-incision torsional phacoemulsification versus biaxial small-incision longitudinal phacoemulsification. J Cataract Refract Surg.

[17] Fernández-Muñoz E, Chávez-Romero Y, Rivero-Gómez R, Aridjis R, Gonzalez-Salinas R (2023). Cumulative dissipated energy (CDE) in three phaco-fragmentation techniques for dense cataract removal. Clin Ophthalmol.

[18] Lundqvist B, Mönestam E (2008). Gender-related differences in cataract surgery outcome: a 5-year follow-up. Acta Ophthalmol.

[19] Sengupta S, Venkatesh R, Krishnamurthy P (2016). Intraocular pressure reduction after phacoemulsification versus manual small-incision cataract surgery: a randomized controlled trial. Ophthalmology.

[20] Elliott DB, Hurst MA, Weatherill J (1990). Comparing clinical tests of visual function in cataract with the patient's perceived visual disability. Eye (Lond).

[21] Colin J, El Kebir S, Eydoux E, Hoang-Xuan T, Rozot P, Weiser M (2010). Assessment of patient satisfaction with outcomes of and ophthalmic care for cataract surgery. J Cataract Refract Surg.

[22] Do VQ, McCluskey P, Palagyi A, White A, Stapleton FJ, Carnt N, Keay L (2018). Patient perspectives of cataract surgery: protocol and baseline findings of a cohort study. Clin Exp Optom.

[23] Schlenker MB, Sayal AP, Yang M, Reid R, Ahmed IIK (2023). Visual acuity, patient-reported outcome measures, or both? The development of an evidence-based appropriateness and prioritization tool for cataract surgery patients. Can J Ophthalmol.

[24] Schlenker MB, Samet S, Lim M, D'Silva C, Reid RJ, Ahmed II (2021). Physician and patient concordance in reporting of appropriateness and prioritization for cataract surgery. PLoS One.

[25] Yong GY, Mohamed-Noor J, Salowi MA, Adnan TH, Zahari M (2022). Risk factors affecting cataract surgery outcome: the Malaysian cataract surgery registry. PLoS One.

[26] Lundström M, Pesudovs K (2011). Questionnaires for measuring cataract surgery outcomes. J Cataract Refract Surg.

[27] Desai P, Reidy A, Minassian DC, Vafidis G, Bolger J (1996). Gains from cataract surgery: visual function and quality of life. Br J Ophthalmol.

[28] Nijkamp MD, Nuijts RM, Borne B, Webers CA, van der Horst F, Hendrikse F (2000). Determinants of patient satisfaction after cataract surgery in 3 settings. J Cataract Refract Surg.

